# Integrating e‐collections following the merger of two specialist hospital libraries: a case study

**DOI:** 10.1111/hir.12305

**Published:** 2020-04-27

**Authors:** Ann Daly, Sandra Harrison, Angela Reed, Derick Yates

**Affiliations:** ^1^ Birmingham Women’s and Children’s NHS Foundation Trust Birmingham UK

**Keywords:** access to information, case studies, collaboration, collection development, e‐journals, libraries, hospital

## Abstract

**Background:**

Birmingham Women’s and Children’s NHS Foundation Trust was formed in February 2017 following an acquisition. The Library and Knowledge Services (LKS) merged while operating across two hospital sites. A priority for the merged Library and Knowledge Service was to integrate e‐collections. A literature review identified six papers reporting health libraries that had merged and integrated e‐collections.

**Objectives:**

A priority for the merged Library and Knowledge Service was to integrate e‐collections.

**Methods:**

To ensure equitable and cost‐effective access to an extended collection, an audit of pre‐existing e‐collections was conducted. Electronic licence agreements enabling cross‐site access were negotiated. A new OpenAthens ID was created.

**Results:**

The integration of e‐collections enabled Trust staff access to a greater number of e‐journals and additional e‐content, and an overall cost‐saving was achieved.

**Discussion:**

This case study supports existing literature stating that integrating collections increases the number of e‐journals. It further identifies cost‐difference in acquiring cross‐site access to e‐journals compared to databases providing full‐text e‐journals and additional e‐content.

**Conclusion:**

Integrating e‐collections enables equity of access and value. A national co‐ordinated approach to procurement of e‐collections will further support equity and best value throughout NHS LKS.

**Key Messages**

- To facilitate equity of access and best value, merged health libraries should integrate e‐collections.
- A journal audit is a valuable tool to identify information need and to integrate e‐collections.
- Health library managers should involve all team members in the integration process to ensure understanding, engagement and shared responsibilities.
- Cost and usage data analysis are important collection development decision aids and can be used for ongoing assessments of value.
- An integrating collections policy should prioritise information need, equity of access and value.

## Background

Birmingham Women’s Hospital delivers care to more than 50 000 women, men and their families every year providing gynaecology, fertility, neonatal, maternity and foetal medicine services. It also accommodates a regional genetic laboratory. Birmingham Children’s Hospital is a specialist paediatric centre caring for children and young people up to 16 years of age, and also provides child and adolescent mental health services. Birmingham Women’s and Children’s (BWC) NHS Foundation Trust was formed in February 2017 following the acquisition of Birmingham Women’s Hospital by Birmingham Children’s Hospital. The merger of the two hospitals enabled more seamless care for women, children and families in Birmingham and the wider region.

The Trust employs approximately 6000 staff with almost 3500 at Birmingham Children’s Hospital, 2000 at Birmingham Women’s and 500 in mental health services, and receives more than 641 000 patient visits per year. The two hospitals are teaching centres, and international centres for research and development working closely with the University of Birmingham, other NHS Hospitals and charitable organisations. In November 2019, the Care Quality Commission awarded the Trust an overall inspection rating of Good.

The Library and Knowledge Services (LKS) also merged in February 2017 while continuing to operate across two hospital sites. Prior to the merger, each LKS had a Library Manager, an Information Specialist and a Senior Library Assistant. Around the time of merger, one Library Manager accepted redundancy and one Specialist left the organisation. Subsequently, two new posts were created: a Senior Library Assistant and a Library Apprentice. The revised cross‐site LKS structure consists of one Library Manager, one Information Specialist and a team of four operational staff. All team members work cross‐site, and since the merger staff training and team building has been a priority.

The merger of the LKS provided impetus to develop a collections policy prioritising information need, equity of access and value while also providing strategic direction and organisational relevance. The integration of e‐collections of the two libraries was organised in line with subscription renewals of January 2018 with preparation starting 4 months earlier. Identifying specialist collections within departments was a challenging part of the process that highlighted the need for a policy statement guiding actions around departmental collections. The integration of e‐collections was a staged process involving all LKS staff to ensure understanding, engagement and shared responsibilities. Regular communication was an important part of the process. The regional OpenAthens Administrator provided advice and support.

This case study starts with a literature review identifying merged health libraries that had integrated e‐collections; six relevant papers were identified. The focus of individual papers varied although each acknowledged that integrating collections and access management were important aspects. This case study also describes methods used to integrate e‐collections at BWC NHS Foundation Trust including an audit of journal holdings and an attempt to identify departmental subscriptions. A single collection was established based on the findings of the audit. This paper outlines processes completed to enable cross‐site access including negotiation of electronic licence agreements and OpenAthens for access management. The paper aims to support NHS LKS undergoing mergers and seeking to integrate e‐journal collections to enable equity of access and best value.

### Literature review

A search for literature reporting on merged health libraries that had integrated their e‐collections was conducted. Databases searched included embase, emerald, health business elite, health management information consortium, the kings fund, lisa, medline and google scholar. These databases were searched from inception to November/December 2018. Search terms used included *health libraries* and *merger* and *e‐journals*. Synonyms, acronyms and variations of these words were used. In addition, the reference lists of included papers were assessed for additional relevant studies. The search retrieved 95 articles with six meeting the inclusion criteria reporting merged health libraries that had integrated e‐collections. Three of these papers reported on merged health libraries in the United States, and the other three in Canada. No papers were located that reported on merged health libraries in the United Kingdom. The focus of individual papers varies from general impact of mergers to consolidating resources and change management. However, all papers acknowledge to some extent that integrating collections and access management are important aspects of library mergers.

Wos and Oddan (1987) provides a Brief Communication detailing a survey to libraries, within multi‐hospital systems in the United States formed from mergers, to determine the effect mergers have on library services including on holdings, budget, staff, collection policies and consortia. The survey reported on 53 libraries and stated, with reference to print journal subscriptions, that 35 libraries held the same number of subscriptions before and after merger while 13 increased subscriptions and five decreased subscriptions. The survey recognised that cooperative collection policies were limited. However, this is unsurprising given the Brief Communication was published 30 years ago when electronic access was uncommon and sharing of collections was therefore limited to print.

The publication of McGowan’s article (2001) and Regenberg, Joyce, Moeller, & Ratner (2002) coincided with the emergence of electronic journals in libraries (Boyce, King, Montgomery, & Tenopir, 2004). It is therefore expected that the authors refer to e‐collections and access management, and come to an agreement that mergers enable a wider choice of e‐resources cross‐site. McGowan (2001) discusses the challenges and successes of the merger of libraries at two hospitals and an ambulatory care centre in Ottawa, Canada and claims that the biggest benefit was access to a larger collection of resources believing this to be a natural and common benefit seen by libraries involved in mergers (2001:154). Regenberg, Joyce, et al. (2002) reports the merger of three hospital libraries and a school of nursing library, and concentrating on the importance of planning and consolidating resources recognising benefits of sharing holdings and of a collective renewal process considering cost, accessibility to full text and usage statistics.

Barnes (2003) provides details of the merger of two hospital libraries in 2001 in Michigan USA; Lansing General Hospital and Ingham Medical Center, an acute care hospital. The focus of the article is management of change and consolidation with details of merging budgets, staffing, acquisitions, interlibrary loan, document supply and literature search services. Barnes (2003) started the merger of the journal collections with development of an inventory of journals following weeding, discarding and archiving of titles. Similar to Wos and Oddan (1987), the collections were primarily print although microfiche collections were also held and these too were merged. Barnes (2003) reports the following:

Among the long term benefits to the consolidation of the libraries are not only cost reduction but higher productivity and greater availability of combined resources to both campuses (2003: 51).

Davis, Shorr, Campbell, & McGowan (2004) uses qualitative and quantitative survey data analysis to review resource management issues relating to the transfer from print textbooks and journals to electronic resources following the merger of three hospital libraries in Ottawa, Canada. The results of the survey indicated that overall users felt that collections had improved or remained satisfactory. However, some users reported confusion with gaining access to e‐resources. In a similar way to Davis, Shorr, et al. (2004), Regenberg, Joyce, et al. (2002) and Barnes (2003) acknowledge that the merger of libraries and integration of collections resulted in more resources, and additionally acknowledges difficulty with e‐access.

The most recent publication is Kenefick and DeVito (2015) that details reasons for merging hospital libraries in the United States, and outlines issues relating to organisational structure, administration, staffing and expectations of library users. Limited information relates directly to merging collections. However, the value of a single interface supporting access is recognised:

… since electronic resource delivery is highly valued it makes sense to devote more time and funds to maintaining current electronic resources and teaching users how to utilise these for evidence based practice. Combining collections with one interface can result in increased electronic resources for all users ( Kenefick & DeVito, 2015: 337).

Kenefick and DeVito (2015) also suggests combining collections may result in a greater number of e‐resources cross‐site and additionally recognises the importance of training users and promoting resources to motivate evidence based practice.

In summary, Wos and Oddan (1987) provides the first published article reporting that mergers had little impact on the number of print journals held. Approximately 15 years later with the emergence of e‐journals, Regenberg, Joyce, et al. (2002), Barnes (2003) and Davis, Shorr, et al. (2004) conclude that library mergers result in an increased number of e‐journals cross‐site and aspects including access management, interface, negotiating licences and cost are important to integrating collections. In more recent years with greater emphasis on access, Kenefick and DeVito (2015) recognises the value of a shared interface to support location and access, and the importance of training users.

This case study supports the findings of the literature review that mergers and integrating collections result in an increased number of journals. It also expands existing literature as it outlines the methods used to integrate collections, and provides details of obtaining electronic licence agreements and organising access management as well as detailing the cost implications of integration.

## Methods

An audit of journal holdings was completed in order to integrate e‐journal collections. There were five stages to the audit:

- Verify journal holdings of individual library sites reporting cost, supplier and usage data
- Identify departmental subscriptions
- Identify duplicate titles between library sites and departments
- Review usage data to identify titles providing good, fair and low value to inform re‐subscription, further promotion or cancellation of title
- Approve a single collection of e‐journals.

### Verify journal holdings

The first stage of the audit verified journal holdings of individual library sites. The scope was e‐journals including single titles and subject‐specific titles available through bibliographic databases. Print journals were outside of the scope of the audit although review of remaining print holdings is an ongoing project.

Individual library sites held an inventory of titles in an Excel spread sheet. The inventories were combined to create a single list of e‐journals that included cost, supplier and usage data. Access to the titles was through an A–Z journal list accessible via the Intranet.

### Identify departmental holdings

The second stage of the audit identified departmental subscriptions. Communications Department sent a global email requesting Trust staff contact the library to discuss locally held subscriptions. Titles identified were added to the inventory.

### Identify duplicate titles

The third stage identified duplicate titles between holdings at Birmingham Women’s LKS, Birmingham Children’s, and individual departments.

### Review usage data

The fourth stage of the audit investigated usage data to identify titles providing value. A value threshold was decided based on cost per download compared to document supply cost that included LKS staff time; cost per download being less than document supply indicated good/fair value, whereas cost per download being more indicated low value. Good value permitted re‐subscription while low value provoked discussion between LKS staff and end‐user and informed promotion or cancellation of title.

### Integrating e‐collections

During the final stage of the audit, a single collection of e‐journals was established with reference to findings of stages 1–4 of the audit.

### Electronic licence agreements and access management

Once the audit was completed, e‐licence and access management were arranged to enable Trust staff at both hospital sites authorised access the newly integrated collection.

### Electronic licence agreements

Journal suppliers were informed of the Trust merger and aim to integrate e‐journal collections, and e‐licence agreements recognising both sites as authorised users were obtained. The e‐licences provided use terms and conditions for access, downloading, document supply and storing. Purchase orders were completed quoting the NICE Electronic and Print Content Framework Agreement and new OpenAthens Organisation Identifier (org ID) recognising the newly merged Trust and certified cross‐site access.

### Access management

Existing access management of e‐journals at both sites was through OpenAthens with two separate org IDs in place. A new single org ID was created to identify the newly merged Trust. Prior to migrating users, a number of OpenAthens housekeeping tasks were completed including identifying and deleting duplicate and expired accounts. Following migration, journal holdings in the Link Resolver were combined.

## Results

See Table 1 for details of holdings and cost pre‐integration of collections, and Table 2 for details of holdings and cost post‐integration. Table 3 provides details of changes to holdings following the audit and Table 4 provides details of costs.

**Table 1:** Holdings and cost pre‐integration of collections

LKS	Holdings	Cost
Birmingham Women’s	22 e‐journals	£18K
Birmingham Children’s	25 e‐journals 2 databases	£27.5K £29.5K
Birmingham Women’s and Children’s	47 e‐journals 2 databases	£45.5K £29.5K
Total cost of holdings pre‐integration		£75K

Pre‐integration of collections, 47 e‐journals and two databases were held between sites costing £75K.

**Table 2:** Holdings and cost post‐integration of collections

LKS	Holdings	Cost
Birmingham Women’s and Children’s	33 e‐journals One database providing 600 + titles and additional e‐content	£29.4K £26.5K
Total cost of holdings post‐integration		£55.9K

Post‐integration of collections, 33 e‐journals were held and one database costing £55.9K.

**Table 3:** Changes to holdings following audit

LKS	Change to holdings following audit
Birmingham Women’s	Four titles cancelled; one available via database, three low usage
Birmingham Children’s	10 titles cancelled; low usage one database cancelled; low usage

13 e‐journals were cancelled due to low usage.

**Table 4:** Cost‐savings and cost‐increase

Details	Cost‐savings	Cost‐increase	Variance
14 no. e‐journal titles cancelled	£15.9K		
1 no. database cancelled	£3K		
1 no. database		£11.5K	
33 no. e‐journals		*£3.6K	
TOTAL	£18.9K	£15.1K	£3.8K

* The £3.6K increase in cost relates to annual inflation.

The cost of enabling cross‐site access to a single database providing access to 600 + e‐journals, healthcare knowledge and additional e‐content was £11.5K; although this was considered to be a substantial increase, it was a negotiated cost that achieved a preferential outcome for the LKS. There was no additional cost to allow cross‐site access to the remaining 33 e‐journal titles. Overall, following the merger and integration of e‐resources, more Trust staff had access to more e‐journals, and a total cost‐saving of £3.8K was achieved.

## Discussion

The audit was a valuable tool to identify and ensure holdings were being managed, and to integrate e‐journal collections. It also identified a departmental collection, and furthermore that departmental staff were unaware of cross‐site holdings. The LKS acquired management of the collection organising procurement and cross‐site access, and continues to work with the department to promote and seek good value. The audit came close to identifying a single departmental title but this was lost to follow‐up with no response to emails.

Additional departmental subscriptions and/or small specialist collections may still exist but have proved challenging to identify. Therefore, LKS teams may benefit from working with finance and procurement departments to identify subscriptions through budget codes and purchase order systems. It is very worthwhile trying to identify departmental holdings as there are a number of advantages of library staff managing all holdings including widening and managing access, monitoring usage to ensure continued value, and supporting compliance with licences and copyright legislation. It is also in the interest of cost‐savings for individual departments and the Trust as a whole.

Cost of cross‐site access to a database was £11.5K; indeed without the cost‐savings through cancellation of titles the LKS budget would have fallen short of meeting the increased cost. However, prior to purchase of a database providing access to health care knowledge including full‐text e‐journals and additional e‐content, an appraisal and cost‐effectiveness study including collection of accurate and meaningful usage data needs to be conducted, and value aligned to judgement of the subject needs of end‐user. The appraisal should include appropriateness of health care knowledge for UK practice, and its evidence base. In addition, the increasing availability of free articles via the Internet and social media, and open access should be considered together with document supply services and cost.

The results of this case study as indicated in Table 4 demonstrate that the cost of sharing e‐collections is dependent on the type of content being shared, for example, clinical database content compared to journal content, rather than being dependent on, for example, size of hospital, number of employees, number of beds, etc. This important information may support library managers in projecting expenditure of integrating collections following a merger. In addition, a nationally co‐ordinated approach to procurement of e‐collections in the context of Knowledge for Healthcare (2014) is progressing and this will further ensure equity and value throughout NHS LKS.

## Conclusion

The integration of e‐collections following a hospital merger was a staged process that included an audit of e‐resources, and involved all LKS staff to ensure understanding, engagement and shared responsibilities. The integration resulted in Trust staff having cross‐site access to a greater number of e‐journals, health care knowledge and additional e‐content, and a cost‐saving was achieved. Promotion and evaluation is an ongoing project for the LKS at BWC to ensure continued value. The variance between cost of acquiring cross‐site access to e‐journals and databases needs consideration and options investigated following a pragmatic appraisal of need and value. Equally, progress with national procurement of e‐collections will enable LKS to further support equity and value.

## Conflict of interest

All authors have no conflict of interest.
